# Low-Frequency Resonant Magnetoelectric Effects in Layered Heterostructures Antiferromagnet-Piezoelectric

**DOI:** 10.3390/s23135901

**Published:** 2023-06-25

**Authors:** Dmitri A. Burdin, Dmitri V. Chashin, Nikolai A. Ekonomov, Leonid Y. Fetisov, Vladimir L. Preobrazhensky, Yuri K. Fetisov

**Affiliations:** 1 MIREA—Russian Technological University, Moscow 119454, Russia; burdin@mirea.ru (D.A.B.); chashin@mirea.ru (D.V.C.); economov@list.ru (N.A.E.); fetisovl@yandex.ru (L.Y.F.); 2 Prokhorov General Physics Institute RAS, Moscow 119991, Russia; vlpreobr@yandex.ru

**Keywords:** magnetoelectric effect, heterostructure, antiferromagnet, hematite, magnetostriction, piezoelectric, magnetic field sensor

## Abstract

Magnetic field sensors using magnetoelectric (ME) effects in planar ferromagnetic-piezoelectric heterostructures convert a magnetic field into an output voltage. The parameters of ME sensors are determined by characteristics of the magnetic constituent. In this work, the low-frequency ME effects in heterostructures comprising a layer of antiferromagnetic hematite α-Fe_2_O_3_ crystal with easy-plane anisotropy and a piezoelectric layer are studied. The effects arise due to a combination of magnetostriction and piezoelectricity because of mechanical coupling of the layers. The field dependences of magnetization and magnetostriction of the hematite crystal are measured. The resonant ME effects in the hematite-piezopolymer and hematite-piezoceramic structures are studied. The strong coupling between magnetic and acoustic subsystems of hematite results in a tuning of the acoustic resonance frequency by the magnetic field. For the hematite layer, the frequency tuning was found to be ~37% with an increase in the bias field up to 600 Oe. For the hematite-PVDF heterostructure, the frequency tuning reached ~24% and the ME coefficient was 58 mV/(Oe∙cm). For the hematite-piezoceramic heterostructure, the frequency tuning was ~4.4% and the ME coefficient 4.8 V/(Oe∙cm). Efficient generation of the second voltage harmonic in the hematite-piezoceramic heterostructure was observed.

## 1. Introduction

In recent decades, magnetic field sensors based on magnetoelectric (ME) effects in composite heterostructures containing ferromagnetic (FM) and piezoelectric (PE) layers have been actively developed and studied. The ME sensors possess a high sensitivity and a large dynamic range, allow for the detection of both permanent and alternating magnetic fields, operate at room temperature, and are simple in design [1,2]. In bulk composite heterostructures with fairly thick layers, ME effects arise because of a combination of magnetostriction of the FM layer and piezoelectricity in the PE layer due to the mechanical coupling of the layers [3,4]. When an alternating magnetic field is applied to the heterostructure, the FM layer is deformed due to magnetostriction, this deformation is transferred to the PE layer, and it generates an alternating electrical voltage (direct ME effect). When the structure is excited by an alternating electric field, the PE layer is deformed due to the inverse piezoelectric effect, the deformation is transferred to the FM layer, which leads to a change in its magnetization (converse ME effect). In magnetic field sensors, the direct ME effect is mostly used.

The characteristics of ME sensors (sensitivity, range of measured fields, noise level, etc.) depend on the magnetic and magnetostrictive properties of the FM layer. To date, ME effects have been studied in detail in structures with layers of metals (Ni, Co), alloys (FeCo, FeGa, amorphous alloys, Terfenol-D) and ferrites (NiFe_2_O_4_, CoFe_2_O_4_), which have a high magnetostriction λ in low magnetic fields [5,6,7]. It has been shown that the magnitude of the ME effect depends on the permanent bias magnetic field *H* applied to the structure [8]. The efficiency of the ME conversion increases by 1–2 orders of magnitude when the excitation field frequency coincides with the frequency of natural acoustic oscillations of the heterostructure due to the resonant increase in deformations [9]. Nonlinear ME effects of generation of harmonics, subharmonics and combination frequencies, the bistability was found with an increasing excitation field [10,11].

In this regard, it is of interest to study ME effects in structures with layers of new materials for ME sensors—antiferromagnets (AFM), whose magnetic, magnetostrictive and acoustic properties differ significantly from the properties of FM layers. Of particular interest are high-temperature AFM single crystals with an easy-plane type anisotropy, which include hematite α-Fe_2_O_3_ with the Neel temperature T_N_ = 960 K and iron borate FeBO_3_ with T_N_ = 348 K. The technologies for growing high-quality crystals of hematite and iron borate are well developed, their magnetic properties have been studied, these crystals are good dielectrics and possess high acoustic quality factors [12,13,14,15,16]. A feature of the AFM crystals with easy-plane anisotropy is a strong coupling of the magnetic and acoustic subsystems, which leads to a tuning of the frequency of acoustic oscillations of crystals by the magnetic field and a nonlinearity of their acoustic characteristics [15,16,17,18].

To the authors’ knowledge, the low-frequency ME effects in composite heterostructures with AFM layers have not yet been studied. The only work published was [19], where a shift in the ferromagnetic resonance frequency under the action of an electric field in the iron borate-piezoelectric heterostructure was observed.

The aim of this work was to study the low-frequency ME effects in heterostructures containing a hematite layer and various piezoelectrics. First, the magnetic, magnetostrictive and magnetoacoustic characteristics of a free hematite plate were measured. Second, the direct resonant ME effect in the structure of hematite-piezopolymer of the PVDF type was investigated. After that, the characteristics of the resonant direct ME effect in the hematite-lead zirconate titanate (PZT) structure were studied. In conclusion, the main results of the work and possibilities of using heterostructures with AFM layers in magnetic field sensors are discussed.

## 2. Materials and Methods

**Figure 1:**
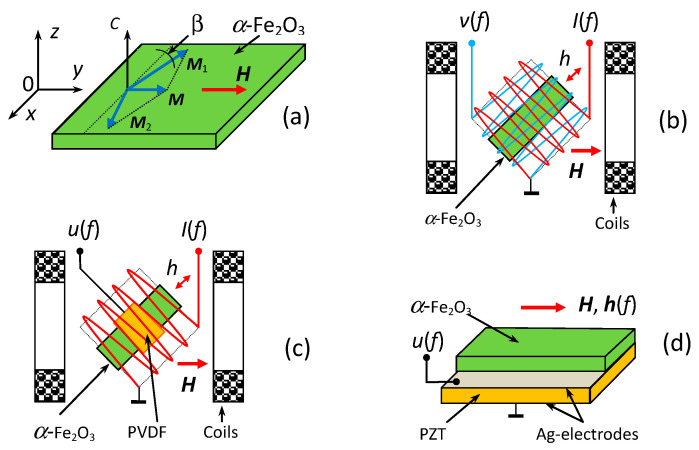
(**a**) Schematic view of magnetic structure of hematite; (**b**) the scheme for excitation and registration of magnetization oscillations in hematite plate; (**c**) the scheme for observation of ME effect in the hematite-PVDF heterostructure; (**d**) schematic view of the hematite-PZT heterostructure.

We used a single crystal of α-Fe_2_O_3_ grown using the method of spontaneous solution-melt crystallization at MIREA by V.A. Murashev [16]. The single crystal was oriented using the X-ray method, cut and polished to optical quality. In measurements, a rectangular plate of hematite with a length of *L* = 17 mm, width of *W* = 5 mm and thickness of *a*_m_ = 0.33 mm was tested. Hematite is a two-sublattice antiferromagnet with easy-plane anisotropy in the temperature range from the Morin temperature *T*_M_ = 260 K to the Neel temperature *T*_N_ = 960 K. Magnetic structure of hematite is schematically shown in Figure 1a. The magnetizations of the sublattices *M*_1_ and *M*_2_ lie in the easy “x-y” plane, the *C*_3_ axis is parallel to the “z” axis, and the binary axis U_2_ is also in the “x-y” plane and directed at the angle β with respect to the field *H*. The sublattices magnetizations are equal to *M*_1_ = *M*_2_ = 870 emu/cm^3^ and canted in a weak external field *H* at an angle φ ≈ 1^0^ with respect to the “y” axis, so that the resulting low magnetization *M* ≈ 2 emu/cm^3^ is directed along the field *H* [20]. The anisotropy in the “x-y” plane is small and ferromagnetic vector *M* can freely rotate in the plane, following the direction of *H*.

The geometry of magnetoacoustic characteristic measurements in a free hematite plate is shown schematically in Figure 1b. The sample was placed inside two flat electromagnetic coils of rectangular cross section, inserted one into the other. The inner coil had dimensions of 20 mm × 20 mm, and the outer one of 23 mm × 23 mm, each containing 50 turns of 0.3 mm wire. The axes of the coils were directed perpendicular to each other. Orthogonal orientation of the coil axes made it possible to minimize the direct electromagnetic pickup from one coil to another and, at the same time, effectively excite magnetization oscillations in the sample. The entire structure was placed between the poles of an electromagnet in an external permanent magnetic field *H* with a strength up to 1.5 kOe. The longitudinal axis of the sample was oriented at an experimentally found angle of ~19^0^ to the direction of the permanent field *H*, at which the magnetization oscillations were most effectively excited. A current with an amplitude up to *I* = 200 mA and a frequency of *f* = 0–500 kHz was passed through the internal coil from an Agilent 33210A generator (Agilent Technologies, Santa Clara, CA, USA), which created an excitation magnetic field with an amplitude *h* up to 2 Oe. Oscillations of the sample magnetization were recorded with an external measuring coil. The dependences of the voltage *V* induced in the measuring coil on the amplitude *h* and frequency *f* of the excitation field *h* and field *H* were measured using an SR844 lock-in amplifier (SRS, Sunnyvale, CA, USA). The permanent magnetic field *H* was measured using a LakeShore 421 Gaussmeter (Lake Shore Cryotronics, Westerville, OH, USA) with an accuracy of 0.1 Oe. The ac excitation field *h* was measured using the current through the coil calibrated at a frequency of 100 Hz. The measuring setup operated in automatic mode under the control of a specialized program.

To study ME effects, two heterostructures were fabricated. The first heterostructure (Figure 1c) contained a hematite plate and a piezoelectric film of poly(vinylidene fluoride) (PVDF-polymer), mounted in the center of the plate with a cyanoacrylate glue. The piezopolymer was chosen as the PE layer because it has low mechanical rigidity and, at the same time, a high piezoelectric-modulus-to-permittivity ratio. The film had in-plane dimensions of 10 mm × 2 mm, thickness of *a*_p_ = 50 μm, piezoelectric modulus of *d*_31_ ≈ 10 pC/N and relative permittivity of ε ≈ 10.4. Ag-electrodes with a thickness of ~1 μm were preliminarily deposited on the film surface using the thermal method. The ME effect was excited by an alternating magnetic field *h*(*f*) produced by the excitation coil. The voltage *u*(*f*) generated by the structure was taken across the electrodes of the PVDF-layer. The amplitude of the ME voltage was recorded at different values of *f*, *h*, and *H*.

The second heterostructure (Figure 1d) contained a hematite plate and a plate of piezoelectric lead-zirconate titanate Pb(Zr_0.52_Ti_0.48_)O_3_ ceramic (PZT). The PZT plate had in-plane dimensions of 17 mm × 6 mm, thickness of *a*_p_ = 250 μm, piezoelectric modulus of *d*_31_ = −175 pC/N and relative permittivity of ε ≈ 1750. The Ag-electrodes with a thickness of ~3 μm were preliminarily deposited on the wafer surface using the firing method. The hematite and PZT layers were bonded with a ~4 μm thick cyanoacrylate adhesive, which provided a strain transfer between the layers. The same setup was used to record the ME voltage *u*(*f*) generated by the PZT layer at different values of *f*, *h* and *H*.

The magnetization curves of the hematite plate *M*(*H*) were measured using a Lakeshore vibrating magnetometer in the field range of *H* = 0–18 kOe with magnetization along the long axis. The field dependence of hematite magnetostriction λ(*H*) was measured using a strain gauge glued to the surface of a hematite plate [21] with an accuracy of δλ ≈ 0.2 × 10^−6^. All measurements were carried out at room temperature without electromagnetic shielding of the structures.

## 3. Results

### 3.1. Magnetization and Magnetostriction of Hematite

**Figure 2:**
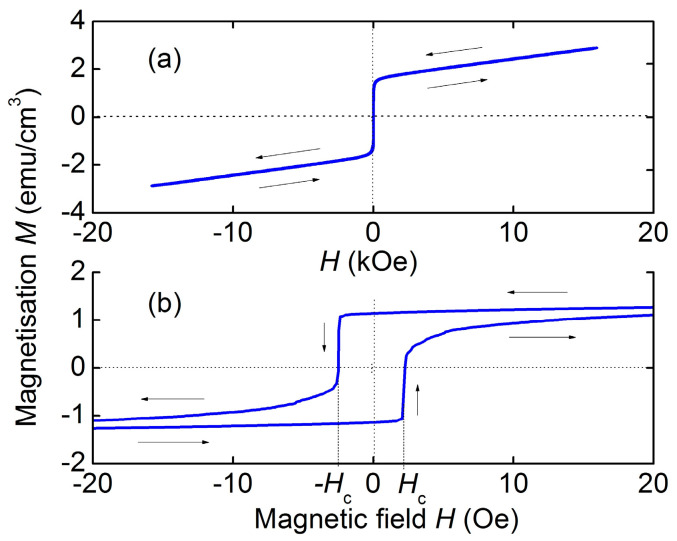
Magnetization curves for the hematite plate: (**a**) in the high field region; (**b**) in the low field region. The arrows show directions of the field change.

Figure 2 shows the magnetization curves for a free α-Fe_2_O_3_ plate when magnetized by field *H* along the long axis. It can be seen from Figure 2a that the magnetization *M* is equal to zero in the region of fields close to zero *H* ≈ 0, then increases abruptly to the value *M* ≈ 2 emu/cm^3^ and grows linearly with a further increase in the field *H* up to 18 kOe. Features of magnetization reversal in the region of low fields with a cyclic change in the field are shown in an expanded scale in Figure 2b. It can be seen that the magnetization *M* begins to grow from zero in the field *H*_c_ ≈ 2 Oe and then smoothly increases with increasing *H*. As the field *H* falls from maximum to zero and its direction changes, a hysteresis occurs. The magnetization drops abruptly in the field *H*_c_ ≈ −2 Oe and the process is repeated. The value of the coercive force was *H*_c_ ≈ 2 Oe.

**Figure 3:**
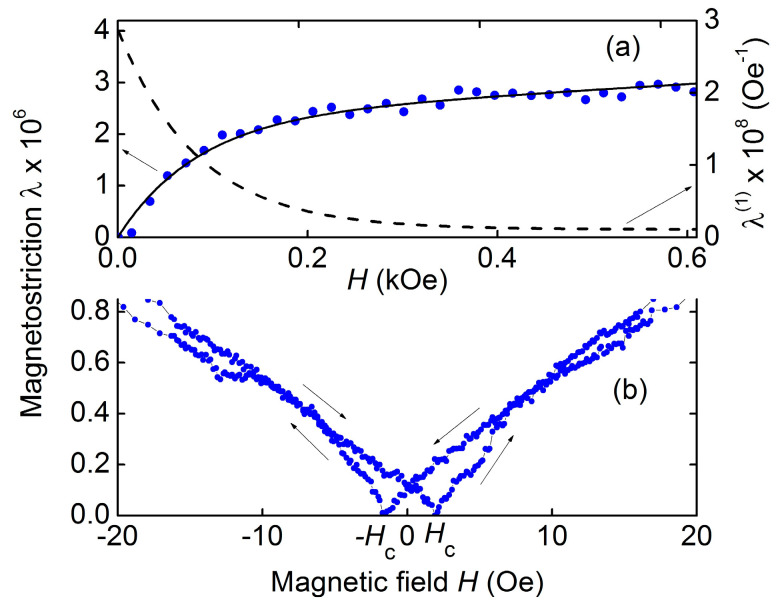
Dependences of hematite magnetostriction λ vs. magnetic field *H*: (**a**) points are the data, solid line is the calculation, dashed line is the piezomagnetic modulus vs. field; (**b**) magnetostriction λ vs. field *H* in the low field region. The arrows show directions of the field change.

Figure 3a shows the dependence of the longitudinal magnetostriction λ(*H*) of the hematite plate on the field *H* applied in the plane along the sample length. The magnetostrictive strain λ first grows from zero to λ_S_ ≈ 2.5 × 10^−6^ with an increase in *H* up to ~300 Oe, and then increases approximately linearly with an increase in the field. Solid and dashed lines in Figure 3a show the calculated field dependences of the magnetostriction λ(*H*) and the piezomagnetic coefficient $\lambda^{\left(1\right)}(H)={\left.\partial \lambda /\partial H\right|}_{H}$, which is equal to the derivative of the magnetostriction with respect to the field. Saturation of the hematite magnetostriction in the range of fields up to 1.5 kOe was not observed.

Figure 3b shows the field dependence of the hematite magnetostriction in the low field region *H* < 20 Oe. It can be seen that the magnetostriction increases approximately linearly with the field and exhibits a hysteresis as the field direction is changed. The coercive force, as for the magnetization curve, was approximately *H*_c_ ≈ 2 Oe.

### 3.2. Frequency Tuning of Acoustic Resonance of Hematite

At the first stage, the magnetoacoustic characteristics of a free hematite plate were studied. Oscillations of the plate magnetization were excited by an alternating harmonic magnetic field *h* created by the first coil, and the output signal *V* was taken from the receiving coil, as shown in Figure 1b.

**Figure 4:**
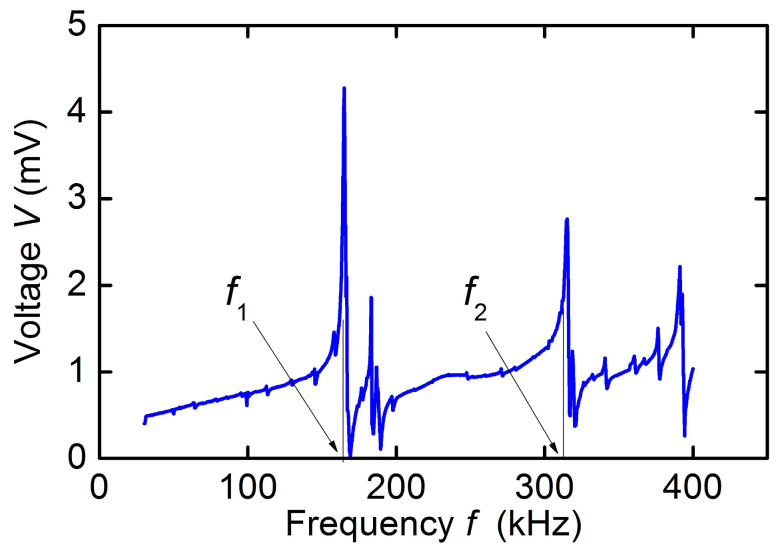
Frequency response of the hematite plate under excitation and registration of acoustic vibrations by coils at excitation field *h* = 0.3 Oe and bias field *H* = 50 Oe.

Figure 4 shows a typical dependence of the voltage amplitude *V* from the receiving coil on the frequency *f* of the excitation field with an amplitude *h* ≈ 0.3 Oe for a hematite plate at *H* = 50 Oe. Several peaks are visible on the characteristic: near the frequency *f*_1_ ≈ 165 kHz with an amplitude of ~4.3 mV and a quality factor *Q*_1_ ≈ 110, near the frequency *f*_2_ ≈ 314 kHz with an amplitude of ~2.7 mV and a quality factor *Q*_2_ ≈ 150, and a peak near the frequency ~394 kHz. The quality factor of the peaks was estimated using the formula *Q* = *f*/Δ*f*, where *f* is the center frequency of the peak, and Δ*f* is the peak width at a level of 0.71 from the maximum. As will be shown later, the peak with frequency *f*_1_ corresponds to the excitation of the fundamental mode of planar vibrations along the length of the plate and the peak with frequency *f*_2_ corresponds to the excitation of the main contour-shear mode of the plate. Other low-amplitude peaks, corresponding to the excitation of higher modes of planar, bending or shear vibrations of the plate will not be considered further.

**Figure 5:**
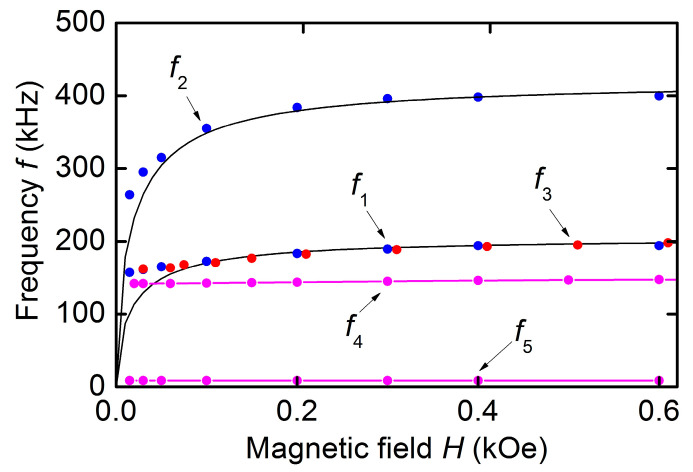
Magnetic field dependences of resonance frequencies *f*_1_ and *f*_2_ for the hematite plate (blue dots), resonance frequency *f*_3_ for the hematite-PVDF heterostructure (red dots), and resonance frequencies *f*_4_ and *f*_5_ for the hematite-PZT heterostructure (purple dots and lines). Points are the data and black solid lines are the calculation using Equation (3).

Figure 5 demonstrates a frequency tuning of the peaks as the field *H* is increased from zero to 0.6 kOe. The relative frequency tuning of resonances of a hematite plate

(1) $$\gamma =\frac{f(\infty )-f(H)}{f(\infty )}100\%$$

where f(∞) is the limiting frequency in high fields corresponding to the layer saturation, and *f*(*H*) is the resonance frequency at field *H*. For the first mode with frequency *f*_1_, the frequency tuning was γ_1_ ≈ 23.4%, and for the second mode with frequency *f*_2_, γ_2_ ≈ 37.1%. When evaluating the frequency tuning, $f_{1}(\infty )$ = 197 kHz was taken for the first mode, and $f_{2}(\infty )$ = 425 kHz for the second mode. Quality factors for both resonances increased from *Q* ≈ 60 in low fields to *Q* ≈ 4 × 10^3^ in the saturation field. The dependences obtained are consistent with the data of [13,22]. Solid lines in Figure 5 show calculated dependences of the peak frequencies on the bias field (see below).

### 3.3. ME Effect in Hematite-PVDF Heterostructure

At the second stage, the frequency and field characteristics of the ME effect in the hematite-PVDF heterostructure were studied. The structure was excited by an alternating magnetic field *h* generated by the excitation coil, and the output voltage *u* was taken across the electrodes of the PVDF layer, as shown in Figure 1c.

**Figure 6:**
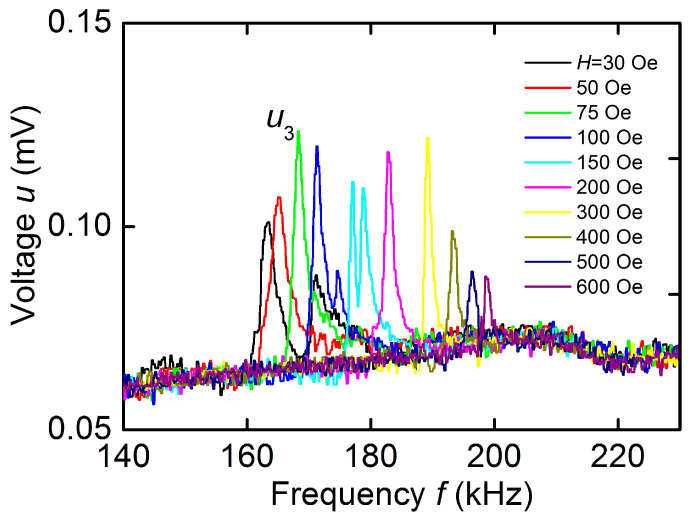
Dependences of the ME voltage *u* on the excitation field frequency *f* for the hematite-PVDF heterostructure at different magnetic fields *H*.

Figure 6 shows the dependences of the voltage *u* on the frequency *f* of the excitation field with an amplitude *h* ≈ 0.2 Oe for different values of the bias field *H*. All the dependences clearly show a resonant peak corresponding to excitation of planar acoustic oscillations of the structure. The peak center frequency *f*_3_ is tuned within ~162–200 kHz with increasing *H*. The peak amplitude *u*_3_ first increases with increasing *H*, reaches a maximum value *u*_3_ ≈ 0.058 mV at magnetic fields *H* = 50–300 Oe, and then decreases gradually as the field increases. The quality factor of the peak in this case increased from *Q* ≈ 50 to ~150. The dependence of the frequency *f*_3_ on the magnetic field *H* plotted using data of Figure 6 is shown in Figure 5. It can be seen that the relative frequency tuning of the acoustic resonance in the hematite-PVDF structure by magnetic field was ~24%. No peaks were observed on the *u*(*f*) characteristic in the high-frequency region of 260–400 kHz.

### 3.4. ME Effect in Hematite-PZT Heterostructure

**Figure 7:**
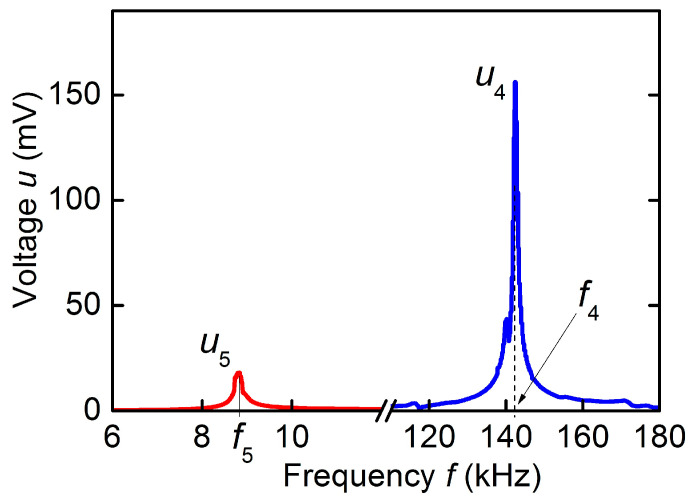
Dependence of the ME voltage *u* on the excitation field frequency *f* for the hematite-PZT heterostructure at excitation field *h* = 1.3 Oe and bias field *H* = 10 Oe. The blue and red peaks correspond to acoustic resonance in the plane of the structure and bending resonance of the structure, respectively.

At the third stage, the characteristics of ME effect in the hematite-PZT heterostructure were studied. Figure 7 shows the dependence of the voltage *u* generated by the PZT layer of the structure on the frequency *f* of the excitation magnetic field for *h* = 1.3 Oe and bias field *H* = 10 Oe. The dependence was measured for orientation of the permanent field *H* and the excitation field *h* along the longitudinal axis of the structure, as shown in Figure 1d.

Two resonances are visible on the curve: a resonance near the frequency *f*_4_ ≈ 142.5 kHz with an amplitude *u*_4_ = 156 mV and a quality factor *Q* ≈ 180, and a weaker resonance near the frequency *f*_5_ ≈ 8.8 kHz with an amplitude *u*_5_ ≈ 18 mV and a quality factor *Q* ≈ 50. The peak with frequency *f*_4_ corresponds to the excitation of the lowest mode of planar vibrations along the length of the heterostructure, and the low-frequency peak with frequency *f*_5_ corresponds to the excitation of bending vibrations of the heterostructure. With an increase in the field *H*, an increase in the frequencies of both peaks was observed. The frequency *f*_4_ increased from 141.5 kHz at *H* = 0 to ~147.8 kHz at *H* = 600 Oe, which corresponds to the frequency tuning by γ ≈ 4.4%. The dependences of the frequencies *f*_4_ and *f*_5_ on the magnetic field *H* for the hematite-PZT heterostructure are shown in Figure 5.

**Figure 8:**
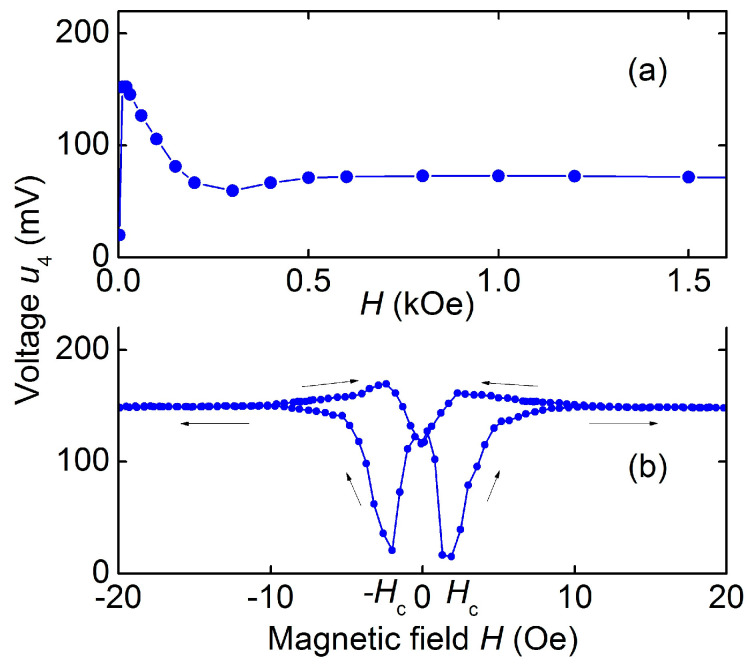
Dependence of the ME voltage *u*_4_ on the magnetic field *H* for the hematite-PZT heterostructure at excitation field *h* = 1.3 Oe and frequency 142 kHz: (**a**) in the wide field region; (**b**) in the low field region. The arrows show directions of the field change.

Figure 8a shows the dependence of the voltage *u*_4_ of the peak with frequency *f*_4_ on the bias field *H*. It can be seen that *u*_4_ first decreases monotonically from the maximum value of ~156 mV at *H* ≈ 0 to ~70 mV at *H* ≈ 0.3 kOe, and then remains approximately constant as the field increases up to 1.5 kOe. One can see a pronounced dip in the region of fields near zero *H* ≈ 0. The fine structure of the dependence *u*_4_(*H*) in the low field region is shown in Figure 8b. The peak amplitude hysteretically depends on the field *H*, reaching a minimum in the fields *H*_min_ ≈ ±2 Oe. In the absence of a field, *H* = 0, the ME voltage *u*_4_ was ~70% of the maximum value at *H* ≈ 2.5 Oe. For a low-frequency resonance with a frequency *f*_5_, the dependences of the voltage *u*_5_ on the magnetic field *H* had the form similar to the dependences shown in Figure 8.

### 3.5. Second Harmonic Generation

With an increase in the excitation field amplitude *h*, the generation of the second voltage harmonic was observed in the hematite-PZT structure. The structure was excited by a field *h* with a frequency equal to half the resonance frequency, and the voltage $u^{\left(2\right)}$ with a doubled frequency, equal to the resonance frequency was recorded. The second harmonic generation was observed only in the region of permanent fields *H* near zero.

**Figure 9:**
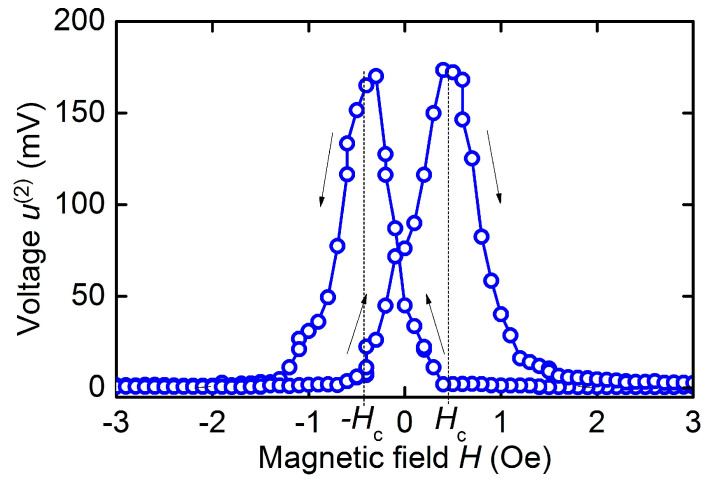
Dependence of the second voltage harmonic $u^{\left(2\right)}$ on permanent field *H* for the hematite-PZT heterostructure at excitation field *h* = 1.3 Oe and frequency 70.85 kHz. The arrows show directions of the field change.

Figure 9 shows the dependence of the second harmonic amplitude $u^{\left(2\right)}$ on the field *H* when the structure is excited by a field with a frequency *f*_1_/2 = 70.8 kHz and an amplitude *h* ≈ 1.3 Oe. One can see a voltage peak with an amplitude of ~170 mV and a width at the base δ*H* ≈ 2 Oe. The peak amplitude and peak width increased approximately linearly with increasing excitation field *h*. Note that the amplitude of the peak is comparable to the amplitude of the voltage generated at linear ME effect (see Figure 7). When generating the second harmonic, there was also a hysteresis on the magnetic field.

## 4. Discussion of Results

First of all, we note the features of magnetization and magnetostriction of a hematite plate, which determine characteristics of ME effects in heterostructures with hematite layers.

As can be seen from Figure 2, the hematite magnetization *M* rapidly increases from zero to *M* ≈ 2 emu/cm^3^ in weak magnetic fields. Using the magneto-optical method, it was shown [23] that in this field region, as *H* increases, the domain structure of the sample is rearranged, leading to the formation of a single-domain state. With a further increase in *H* up to 20 kOe, the magnetization grows linearly with the field due to the canting of the sublattices magnetizations in the field direction. In the fields ranging up to several kOe, the resulting magnetization of hematite *M* is small. Therefore, the demagnetization effects are also small and should not affect the characteristics of ME effects. Consequently, the field characteristics of ME effects in heterostructures with hematite layers will not depend on the layers size, in contrast to heterostructures with FM layers, where demagnetization effects play a significant role [24,25].

The field dependence of the hematite magnetostriction λ(*H*), as can be seen from Figure 3a, differs qualitatively from the typical field dependence of magnetostriction of FM materials used in ME heterostructures. In low fields, λ of hematite grows approximately linearly with the field (not quadratically, as in ferromagnets), then its growth rate slows down. In high fields, λ again grows linearly with increasing field, but more slowly. =A similar dependence λ(*H*) for hematite was observed in [26,27], where it was also shown that value of the magnetostriction in high fields depends on the sample shape and orientation of the field *H*.

The measured field dependence of hematite magnetostriction is described by

(2) $$\lambda (H)=\lambda_{S}(\infty )\left[1-e^{-\alpha H}\right]+\tau H$$

where the experimentally found parameters *λ_S_* = 2.31 × 10^−6^, *α* = 0.012 Oe^−1^ and *τ* = 1.1 × 10^−3^ Oe^−1^ are taken. The calculated curve is shown with a solid line in Figure 3a. The dashed line in Figure 3a shows the field dependence of the piezomagnetic coefficient $\lambda^{\left(1\right)}(H)$, found by differentiating the function (2). The maximum coefficient was ~2.8 × 10^−8^ Oe^−1^, i.e., an order of magnitude smaller than for Metglas [10].

A unique property of antiferromagnets with easy-plane anisotropy is a giant frequency tuning of acoustic resonance by magnetic field, which occurs due to the strong coupling of magnetic and acoustic subsystems of the material [17,18]. The magnetoelastic coupling leads to a renormalization of elastic moduli of the material followed by a magnetic field dependence of the acoustic resonance frequencies of the sample—the so-called delta-*E* effect. The dependence of the resonance frequency *f* on the field *H* is given by the following formula [18]:

(3) $$f(H)=f_{0}(\infty )\sqrt{1-\frac{2H_{E}H_{ms}(\beta )}{H(H+H_{D})+2H_{E}H_{ms}}}$$

where $f_{0}(\infty )$ is the maximum frequency corresponding to the saturated magnetoelastic coupling, *H*_D_ = 22 kOe is the Dzyaloshinski field, *H*_E_ = 9.2 × 10^3^ kOe is the effective exchange field [15], and *H*_ms_ is the magnetostriction field. Here, $H_{ms}(\beta )=H_{ms}\cos^{2}(2\beta )$ is for the contour-shear mode and $H_{ms}(\beta )=H_{ms}\sin^{2}(2\beta )$ is for the longitudinal mode, where β is the angle between magnetic field *H* and the binary axis U_2_.

The dependences of the resonance frequencies *f*_1_ and *f*_2_ on the field *H* calculated using Equation (3) are shown with solid lines in Figure 5. The parameters of the hematite plate found from experiment 2*H*_E_*H*_ms_ ≈ 2.2 kOe^2^, $f_{1}(\infty )$ = 197 kHz, $f_{2}(\infty )$ = 425 kHz, and angle β = 19^0^ were used in calculations. The limiting frequencies can be estimated by the formulas: $f_{1}(H=\infty )=(1/2L)\sqrt{Y/\rho }$ for planar vibrations along the plate length *L* and $f_{2}(H=\infty )=(1/2W)\sqrt{C_{66}/\rho }$ for contour-shear vibrations along the plate width *W*. Calculation for a hematite plate of length *L* = 17 mm, width *W* = 5 mm, Young’s modulus *Y* = 23 × 10^10^ N/m^2^, and shear modulus *C*_66_ = 9.3 × 10^10^ N/m^2^ [13], gives the frequencies $f_{1cal}(\infty )=$ 193 kHz and $f_{2\mathrm{cal}}(\infty )=$ 419 kHz, respectively. Thus, the theory explains the field dependences of the resonance frequencies of hematite plate well.

For the hematite-PVDF heterostructure, the field dependence of the resonance frequency *f*_3_(*H*), as can be seen from Figure 3, completely coincided with the dependence *f*_1_(*H*) for a free hematite plate. This indicates that a thin PVDF film deposited on the hematite surface did not affect its magnetoacoustic characteristics. The frequency tuning of the resonant ME effect under magnetic field in the structure under study was ~24%, i.e., an order of magnitude greater than the frequency tuning due to the delta-E effect in structures with various FM materials: 1% in the structure with permendure (FeCoV) [5], 1.4% in the structure with amorphous FeGaB alloy [28], and 3.9% in the structure with Terfenol-D [29]. The maximum value of ME voltage coefficient for the hematite-PVDF heterostructure, as follows from data in Figure 6 was $\alpha_{E}=u_{3}/(a_{p}h)\approx$ 58 mV/(Oe∙cm) at a bias field of *H* ≈ 75–100 Oe. It is seen from Figure 6 that the resonant peak splits in magnetic fields of *H* ~ 100–150 Oe. This may be due to the intersection at a given field of the dispersion curves of vibration modes with close frequencies, which was recently observed in hematite disk resonators [22]. A lack of ME effect in the hematite-PVDF heterostructure at the frequency of contour-shear vibrations *f*_2_ is explained by the non-responsivity of the PVDF film to shear deformations.

It is seen from Figure 5, that the resonance frequency *f*_4_ of the hematite-PZT heterostructure increased by 4.5% with increasing *H*, i.e., the frequency tuning decreased by a factor of ~6 compared to the frequency tuning for a free hematite plate. The mechanism of magnetoelastic excitation in the geometry of Figure 1d is modulation of the non-saturated magnetostriction by the longitudinal alternating magnetic field. Longitudinal susceptibility in the fields up to 1.5 kOe is mainly determined by the residual growth stresses and weakly dependents on magnetizing field. As a result, the sensitivity of the magnetoelastic coupling to the magnetic field variations also deceases.

The limiting frequencies of the lowest modes of planar *f*_4_ and bending *f*_5_ resonances for the hematite-PZT structure were estimated using the formulas for the natural vibration frequencies of a free rod [30]. Taking into account dimensions of the structure, effective values of Young’s modulus *Y*_ef_ = 16.1 × 10^10^ N/m^2^ and density $\rho_{ef}=$ 6.2 × 10^3^ kg/m^3^, the frequencies *f*_4cal_ ≈ 149.2 kHz and *f*_5cal_ ≈ 8.02 kHz were obtained, which are in good agreement with the measured ones. The maximum value of the ME voltage coefficient for the hematite-PZT heterostructure, as follows from the data in Figure 8, was $\alpha_{E}=u_{4}/(a_{p}h)\approx$4.8 V/(Oe∙cm) at a bias field of *H* ≈ 2.5 Oe.

It can be seen from Figure 8a that the field dependence of ME voltage *u*_4_(*H*) for the hematite-PZT heterostructure qualitatively differs from similar dependence for the structures with FM layers. This is due to the unusual field dependence of the magnetostriction λ(*H*) and piezomagnetic coefficient $\lambda^{\left(1\right)}(H)$ of hematite, which are shown in Figure 3a. It is known that in composite heterostructures with a stress-mediated ME effect, the dependence *u*(*H*) qualitatively repeats field dependence of the piezomagnetic modulus of magnetic layer [4]. Comparison of the curves $\lambda^{\left(1\right)}(H)$ in Figure 3a and *u*_4_(*H*) in Figure 8a confirms this connection. The quantitative difference between the experiment and theory can be due to a change in the shape of the dependence λ(*H*) for a hematite plate loaded with a PZT layer.

Finally, we note the high efficiency of the second voltage harmonic generation in the hematite-PZT heterostructure. Using the data in Figure 9, we obtain a nonlinear ME coefficient $\alpha_{E}^{\left(2\right)}=u^{\left(2\right)}/(a_{p}h^{2})\approx$ 4 V/(cm∙Oe^2^), which is comparable to the coefficient for the Metglas-PZT structure ~4.5 V/(cm∙Oe^2^) and exceeds by an order of magnitude the coefficients for structures with Ni or FeCo layers [31]. The high nonlinearity of ME effect is due to peculiarities of the hematite magnetostriction: the linear field dependence of the magnetostriction in low fields *H* ≈ 0 and symmetry of the magnetostriction with respect to the field direction λ(*H*) = λ(−*H*) (see Figure 3b). Therefore, the amplitude of the second harmonic at *H* ≈ 0 is proportional to the magnetostriction *u*^(2)^~λ, and not to its second derivative $u^{\left(2\right)}\sim \lambda^{\left(2\right)}$, as for the structures with ferromagnetic layers. The amplitude *u*^(2)^ should be maximum at *H* = 0 and drop to zero with increasing magnetic field up to *H* ≈ *h*, which was observed experimentally. A decrease in the hysteresis of the nonlinear ME effect during second harmonic generation (see Figure 9) down to *H*_c_ ≈ 0.5 Oe compared to the hysteresis of linear ME effect (Figure 8b) may be due to suppression of ME effect hysteresis with an increase in the amplitude of the excitation magnetic field [32].

The above features of ME effects in heterostructures with hematite layers make it possible to expand the functionality of magnetic field sensors. In particular, magnetic tuning of the resonant frequency of the hematite-PVDF and hematite-PZT heterostructures can be used to fine-tune the sensors to the frequency of the measured alternating magnetic field. The dependence of the acoustic resonance frequency of heterostructures on the field makes it possible to elaborate self-oscillating permanent magnetic field sensors with a frequency output. The unambiguous dependence of ME voltage at the resonance frequency on the field in the hematite-PZT heterostructure can be used in sensors of permanent magnetic fields. The strong nonlinearity of the heterostructures allows for the realization of frequency doublers operating without a bias field.

## 5. Conclusions

Thus, we observed and investigated the direct resonant ME effect in heterostructures with a magnetostrictive layer of antiferromagnetic hematite α-Fe_2_O_3_ single crystal and piezoelectric layers of PVDF polymer or PZT piezoceramics. The dependences of the magnetization *M* and magnetostriction λ of a hematite plate on the permanent magnetic field are measured. It is shown that the strong coupling of magnetic and acoustic subsystems in the hematite crystal leads to a change in its rigidity (delta-E effect) and allows one magnetic tuning of the acoustic resonance frequency of crystals up to ~37%. In the hematite-PVDF heterostructure, the frequency tuning of planar acoustic resonance by magnetic field reached 24%, and the value of ME coefficient was 58 mV/(Oe∙cm). In the hematite-PZT heterostructure, the resonance frequency tuning by magnetic field reached ~4.4%. The ME coefficient in weak magnetic fields was ~4.8 V/(Oe∙cm) and monotonically decreased with increasing field. Efficient generation of the second voltage harmonic in the hematite-piezoceramic heterostructure in the absence of a bias field was found. The results show that by choosing the material and the size of the PE layer in bilayers with hematite, it is possible to realize both a wideband magnetic tuning of the resonance frequency and a high efficiency of ME conversion. The ME effects in heterostructures with layers of antiferromagnetic hematite single crystals open up new possibilities for creating magnetic field sensors.

## Data Availability

The data that support the findings of this study are available from the corresponding author upon reasonable request.

## References

[1] Gao J., Jiang Z., Zhang S., Mao Z., Shen Y., Chu Z. (2021). Review of magnetoelectric sensors. Actuators.

[2] Kumar A., Kaur D. (2022). Magnetoelectric heterostructures for next-generation MEMS magnetic field sensing applications. J. Alloys Compd..

[3] Van Suchtelen J. (1972). Product properties: A new application of composite materials. Philips Res. Rep..

[4] Nan C.-W., Bichurin M.I., Dong S., Viehland D., Srinivasan G. (2008). Multiferroic magnetoelectric composites: Historical perspective, status, and future directions. J. Appl. Phys..

[5] Filippov D.A., Laletin V.M., Poddubnaya N.N., Galichyan A., Zhang J., Srinivasan G. (2020). Magnetoelectric and magnetostriction characteristics of symmetric three layered structures of nickel-lead zirconate titanate–nickel and permendure–lead zirconate titanate–permendure. IOP Conf. Ser. Mater. Sci. Eng..

[6] Liang X., Dong C., Chen H., Wang J., Wei Y., Zaeimbashi M., He Y., Matyushov A., Sun C., Sun N. (2020). A Review of thin-film magnetoelastic materials for magnetoelectric applications. Sensors.

[7] Dong S., Zhai J., Bai F., Li J., Viehland D., Lograsso T.A. (2015). Magnetostrictive and magnetoelectric behavior of Fe–20at.% Ga/Pb(ZrTi)O_3_ laminates. J. Appl. Phys..

[8] Srinivasan G., De Vreugd C.P., Laletin V.M., Paddubnaya N., Bichurin M.I., Petrov V.M., Filippov D.A. (2005). Resonant magnetoelectric coupling in trilayers of ferromagnetic alloy and piezoelectric lead zirconate titanate: The influence of bias magnetic field. Phys. Rev. B.

[9] Bichurin M.I., Filippov D.A., Petrov V.M., Laletsin V.M., Paddubnaya N., Srinivasan G. (2003). Resonance magnetoelectric effects in layered magnetostrictive-piezoelectric composites. Phys. Rev. B.

[10] Fetisov L.Y., Burdin D.A., Ekonomov N.A., Chashin D.V., Zhang J., Srinivasan G., Fetisov Y.K. (2018). Nonlinear magnetoelectric effects at high magnetic field amplitudes in composite multiferroics. J. Phys. D Appl. Phys..

[11] Fetisov Y.K., Burdin D.A., Ekonomov N.A., Fetisov L.Y., Berzin A.A., Hyes P., Quandt E. (2018). Bistability in a multiferroic composite resonator. Appl. Phys. Lett..

[12] Morrish A.H. (1995). Chapter 2—Canted antiferromagnetism: Hematite. Crystal Growth and Characterization of Hematite.

[13] Andruschak E.A., Evtikhiev N.N., Pogozhev S.A., Preobrazhensky V.L., Ekonomov N.A. (1981). Acoustic vibrations in antiferromagnetic resonators. Sov. Phys. Acoust..

[14] Dubenko I.S., Kiselev A.O., Murashov V.A., Preobrazhensky V.L. (1991). Influence of impurities and technology on thermostability of magneto-acoustic parameters of hematite. Non-Org. Mater..

[15] Strugatsky M.B., Skibinsky K.M. (2007). Acoustic resonances in antiferromagnet FeBO_3_. J. Magn. Magn. Mater..

[16] Evdokimov A.A., Murashov V.A., Preobrazhensky V.L., Tolksdorf W. (1983). Growth and magnetoacoustic properties of hematite crystals. Crystal Growth 1983: Proceedings of the Seventh International Conference on Crystal Growth, Stuttgart, Germany, 12–16 September 1983.

[17] Seavey M.H. (1972). Acoustic resonance in the easy-plane weak ferromagnets α-Fe_2_O_3_ and FeBO_3_. Solid State Commun..

[18] Ozhogin V.I., Preobrazhenskii V.L. (1988). Anharmonicity of mixed modes and giant acoustic nonlinearity of antiferromagnetics. Sov. Phys. Uspekhi.

[19] Popov M., Liu Y.V., Safonov L., Zavislyak I.V., Moiseienko V., Zhou P., Fu J., Zhang W., Zhang J., Qi Y. (2020). Strong converse magnetoelectric effect in a composite of weakly ferromagnetic iron borate and ferroelectric lead zirconate titanate. Phys. Rev. Appl..

[20] Dzyaloshinsky I. (1958). A Thermodynamic theory of “weak” ferromagnetism of antiferromagnetics. J. Phys. Chem. Solids.

[21] Chashin D.V., Burdin D.A., Fetisov L.Y., Ekonomov N.A., Fetisov Y.K. (2018). Precise measurements of magnetostriction of ferromagnetic plates. J. Sib. Fed. Univ. Math. Phys..

[22] Moshkin V., Preobrazhensky V., Pernod P. (2020). Wide-range frequency control in magnetoacoustic resonator. IEEE Trans.Ultrason. Ferroelectr. Freq. Control.

[23] Preobrazhenskii V.L., Shishkov A.A., Ekonomov N.A. (1987). Magnetooptic registration of domain structure of hematite. Solid State Phys..

[24] Lasheras A., Gutiérrez J., Barandiarán J.M. (2016). Quantification of size effects in the magnetoelectric response of metallic glass/PVDF laminates. Appl. Phys. Lett..

[25] Fedulov F.A., Saveliev D.V., Chashin D.V., Shishkin V.I., Fetisov Y.K. (2022). Magnetoelectric effects in stripe- and periodic heterostructures based on nickel–lead zirconate titanate bilayers. Russ. Technol. J..

[26] Urquhar H.M.A., Goldman J.E. (1956). Magnetostrictive effects in an antiferromagnetic hematite crystal. Phys. Rev..

[27] Voskanyan R.A., Levitin R.Z., Shchurov V.A. (1968). Magnetostriction of a hematite monocrystal in fields up to 150 kOe. Sov. Phys. JETP.

[28] Li M., Matyushov A., Dong C., Chen H., Lin H., Nan T., Qian Z., Rinaldi M., Lin Y., Sun N.X. (2017). Ultra-sensitive NEMS magnetoelectric sensor for picotesla DC magnetic field detection. Appl. Phys. Lett..

[29] Lei C., Ping L., Yu-Mei W., Yong Z. (2013). Tunable characteristics of bending resonance frequency in magnetoelectric laminated composites. Chin. Phys. B.

[30] Timoshenko S. (1955). Vibration Problems in Engineering.

[31] Burdin D.A., Chashin D.V., Ekonomov N.A., Fetisov L.Y., Fetisov Y.K., Srinivasan G., Sreenivasulu G. (2014). Nonlinear magnetoelectric effects in planar ferromagnetic-piezoelectric structures. J. Magn. Magn. Mater..

[32] Burdin D.A., Chashin D.V., Ekonomov N.A., Fetisov L.Y., Fetisov Y.K. (2018). Suppression of nonlinear magnetoelectric effect hysteresis in a layered ferromagnetic-piezoelectric structure. J. Magn. Magn. Mater..

